# Loss of miR-1258 contributes to carcinogenesis and progression of liver cancer through targeting CDC28 protein kinase regulatory subunit 1B

**DOI:** 10.18632/oncotarget.9728

**Published:** 2016-05-30

**Authors:** Minghua Hu, Mingwei Wang, Huihong Lu, Xiaoming Wang, Xiaoshan Fang, Jinguo Wang, Chenyang Ma, Xiaobing Chen, Hongping Xia

**Affiliations:** ^1^ Department of Hepatobiliary Surgery, Affiliated Yijishan Hospital of Wannan Medical College, Wuhu, 241001, China; ^2^ Division of Cardiovascular Medicine, The Affiliated Hospital of Hangzhou Normal University, Hangzhou, 310015, China; ^3^ Department of Oncology, The Affiliated Cancer Hospital, Zhengzhou University, Zhengzhou, 450008, China; ^4^ Department of Anesthesiology, East Hospital, Tongji University School of Medicine, Shanghai, 200120, China

**Keywords:** miR-1258, liver cancer, recurrence, stemness, CKS1B

## Abstract

Hepatocellular carcinoma (HCC) is the leading cause of cancer related death worldwide. The number of deaths is proportional to the global incidence, which highlights the aggressive tumor biology and lack of effective therapies. Dysregulation of microRNAs has been implicated in carcinogenesis and progression of liver cancer. Here, we identified that miR-1258 was significantly downregulated in HCC and associated with poor patients' survival. Overexpression of miR-1258 significantly inhibits liver cancer cell growth, proliferation and tumorigenicity through increasing cell cycle arrest in G0/G1 phase and promotes cell apoptosis. Interestingly, stable overexpression of miR-1258 suppresses cell migration, stemness and increases sensitivity of HCC cells to chemotherapy drug like doxorubicin. The CDC28 protein kinase regulatory subunit 1B (CKS1B) was identified as a functional downstream target of miR-1258. Re-expression of CKS1B overcomes miR-1258 induced apoptosis and increases stemness of HCC cells, suggesting that loss of miR-1258 contributes to carcinogenesis and progression of liver cancer through targeting CKS1B. Therefore, loss of miR-1258 may be a potential diagnostic and prognostic biomarker and blocking miR-1258-CKS1B axis is a potential therapeutic strategy in HCC.

## INTRODUCTION

Hepatocellular carcinoma (HCC) is the most common type of primary liver cancer (70%–90%) and is seen more often in men than in women. It is the second leading cause of cancer death among men in developing countries and the sixth leading cause of cancer death among men in developed countries. An estimated 782,500 new liver cancer cases and 745,500 deaths occurred worldwide during 2012, with China alone accounting for about 50% of the total number of cases and deaths [1]. The current management of HCC patients includes surgical resection, liver transplantation, radiofrequency ablation, transcatheter arterial chemoembolization (TACE) or sorafenib. Unfortunately, most patients are still diagnosed at advanced disease stages, and in this setting, sorafenib is currently the only FDA approved targeted therapy drug to improve overall survival. Even if after curative resection in patients with early-stage disease, tumour recurrence is estimated to occur in 70% of patients. The adjuvant sorafenib for HCC after resection or ablation (STORM) is a phase 3, randomised, double-blind, placebo-controlled trial, which indicated that sorafenib is not an effective intervention in the adjuvant setting for HCC following resection or ablation [2]. Thus, the study of novel factors contributing to carcinogenesis and progression of liver cancer needs to be investigated.

Recently, microRNAs (miRNAs), a kind of small non-coding RNAs expressed in different tissue and cell types, are critical regulator of innumerous biological processes that suppress the expression of target genes. The dysregulated miRNA expression has been correlated with many human diseases including HCC. Over the past few years, increasing studies have evaluated the role of miRNAs in hepatocarcinogenesis and tumor progression. The diagnostic, prognostic, and therapeutic potential of miRNA has been shown in HCC [3, 4]. Studies comparing the microRNA profiles of diseased and normal tissues from patients with HCC have revealed consistent changes in the expression of many miRNAs. As a result, genes regulated by these miRNAs are also abnormally expressed, which can contribute to the development of HCC. Through combination miRNA expression profile of liver HCC (LIHC) from The Cancer Genome Atlas (TCGA) and the other HCC miRNA expression profile dataset (GSE36915). Many consistently dysregulated miRNAs in HCC were identified. Among them, the dysregulation and role of miR-1258 in HCC has not been investigated.

In this study, we found that miR-1258 was significantly downregulated in HCC and associated with poor patients' survival. Overexpression of miR-1258 significantly inhibits the expression of CDC28 protein kinase regulatory subunit 1B (CKS1B) and overcomes oncogenic properties *in vitro* and tumorigenicity *in vivo* of HCC cells. This study suggested that loss of miR-1258 correlates to the expression level of CKS1B to influence the carcinogenesis of HCC and is likely to become the key strategy to the treatment of HCC.

## RESULTS

### miR-1258 was significantly downregulated in HCC and associated with patients' survival

**Figure 1:**
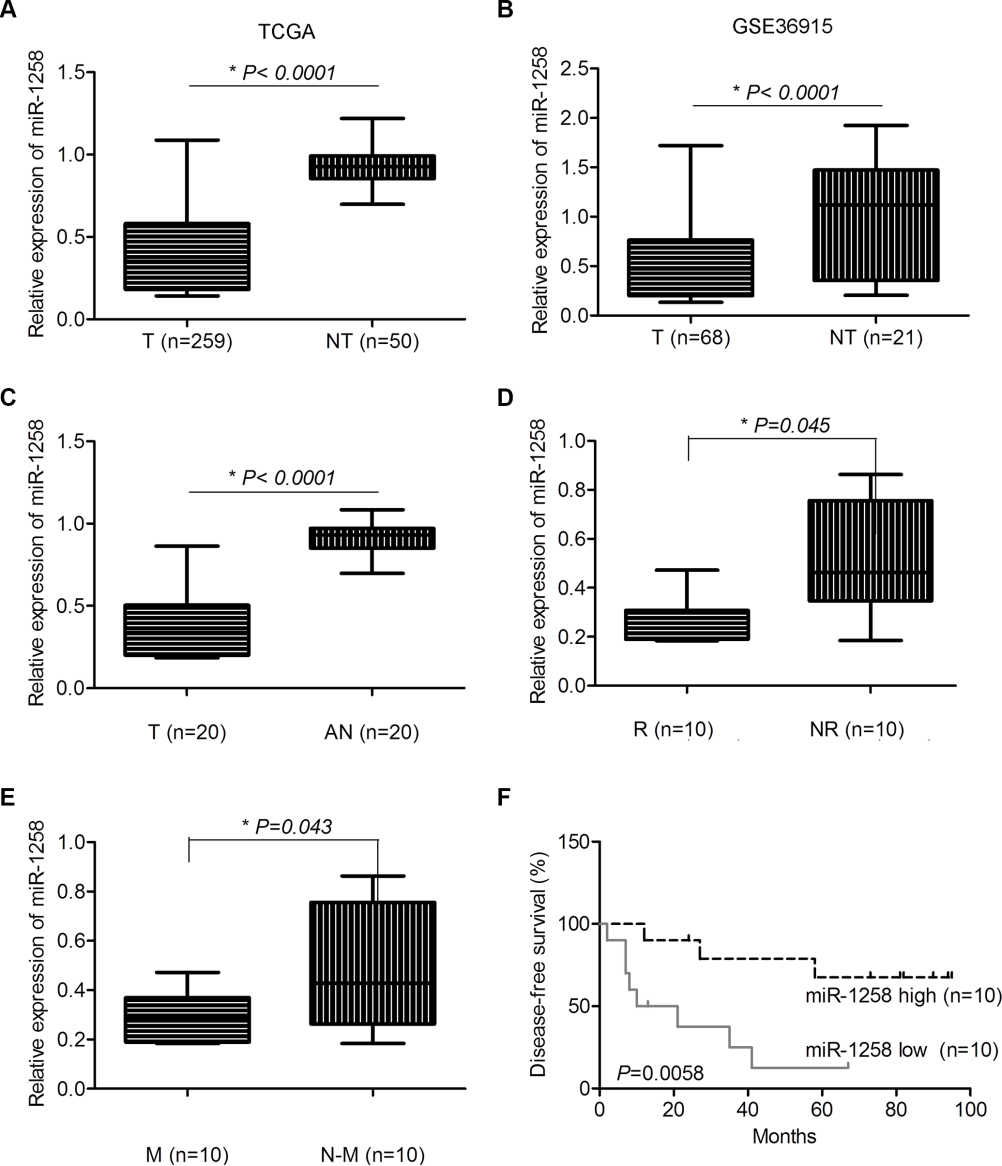
miR-1258 was significantly downregulated in HCC and associated with patients' survival (**A** and **B**) The normalized relative expression level of miR-1258 in HCC tumor and non-tumor samples in TCGA (A) and GSE36915 dataset (B) (T: Tumor, NT: Non-Tumor). (**C**) The normalized relative expression of miR-1258 in 20 pairs of HCC and adjacent normal samples by RT-qPCR analysis (T: Tumor, AN: Adjacent Normal). (**D** and **E**) The expression of miR-1258 was associated with tumor recurrence (D) and metastasis (E) (R: Recurrence, NR: Non-Recurrence, M: Metastasis, N-M: Non-Metastasis). (**F**) The expression of miR-1258 was significantly associated with patients' survival.

To identify the consistently dysregulated miRNAs in HCC, we combined the miRNA expression profile of LIHC from TCGA and the other HCC miRNA expression profile dataset (GSE36915), which has miRNA expression profile of 68 HCC and 21 non-tumor liver tissues. A panel of consistently dysregulated miRNAs were identified in both datasets. Among them, the downregulation of miR-1258 was consistently observed in both TCGA (Figure 1A) and GSE36915 (Figure 1B). We further validated several consistently dysregulated miRNAs of TCGA and GSE36915 in 20 pair of HCC tissues with recurrence and metastasis information by real time quantitative reverse transcription PCR (RT-qPCR). The results showed that the expression of miR-1258 was significantly downregulated in HCC tissues compared to adjacent normal samples (Figure 1C). Downregulation of miR-1258 in HCC was also shown to be associated with tumor recurrence and metastasis (Figure 1D and 1E). Furthermore, when survival analysis was performed using the median expression values of miR-1258, low expression of miR- 1258 in HCC was significantly associated with poor disease-free survival (Figure 1F). These clinical expression data suggested that loss of miR-1258 may contribute to the development of HCC.

### Overexpression of miR-1258 inhibits liver cancer cell growth and proliferation

**Figure 2:**
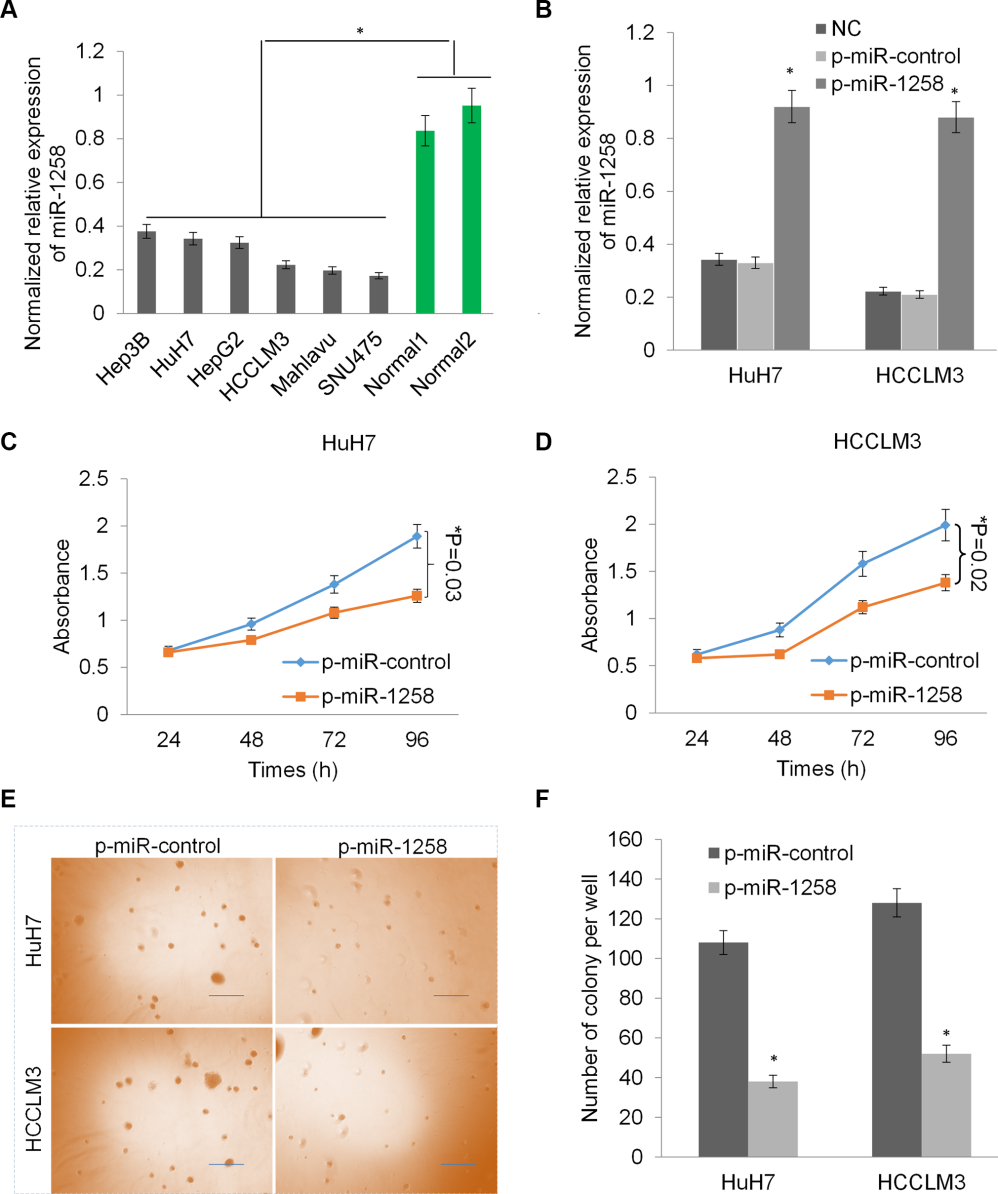
Overexpression of miR-1258 inhibits liver cancer cell growth and proliferation (**A**) The normalized relative expression of miR-1258 in a panel of liver cancer cells and two normal liver tissues by RT-qPCR analysis. (**B**) The expression of miR- 1258 was significantly increased in HuH7 and HCCLM3 cells transfected with p-miR-1258 plasmid compare with p-miR-control plasmid transfection or normal control (NC). (**C** and **D**) Overexpression of miR-1258 in HuH7 (C) and HCCLM3 (D) significantly inhibits liver cancer cells growth by MTS analysis. (**E**) The represent images of soft agar colony formation in HuH7 and HCCLM3 cell transfected with p-miR-1258 or p-miR-control. (**F**) The number of colonies formed in in HuH7 and HCCLM3 cell transfected with p-miR-1258 or p-miR- control was counted under the microscope. All data are given as mean ± SD of three independent experiments. Significant differences are indicated, *P < 0.05.

Consistently, the downregulation of miR-1258 was also observed in a panel of liver cancer cell lines (Figure 2A). To investigate the critical role of miR-1258, we stably transfected two HCC cells with p-miR-1258 or p-miR-control plasmid to overexpress miR-1258. The RT-qPCR analysis showed that the expression of miR- 1258 was significantly increased in the selected stably transfected cells compared to untransfected control cells or p-miR-control plasmid stably transfected cells (Figure 2B). The cell growth was dramatically reduced by overexpression of miR-1258 in HuH7 and HCCLM3 cells (*P < 0.05, Figure 2C and 2D). Overexpression of miR-1258 also significantly inhibit the colony formation ability of HuH7 and HCCLM3 in soft agar, suggesting that miR-1258 inhibits cellular anchorage-independent growth of HCC cells *in vitro* (Figure 2E and 2F). Thus, these data revealed that loss of miR-1258 in HCC promotes tumor cell growth and proliferation and re- expression of miR- 1258 inhibits liver cancer cell growth and proliferation.

### miR-1258 induces cell cycle arrest in G0/G1 phase and promotes cell apoptosis

**Figure 3:**
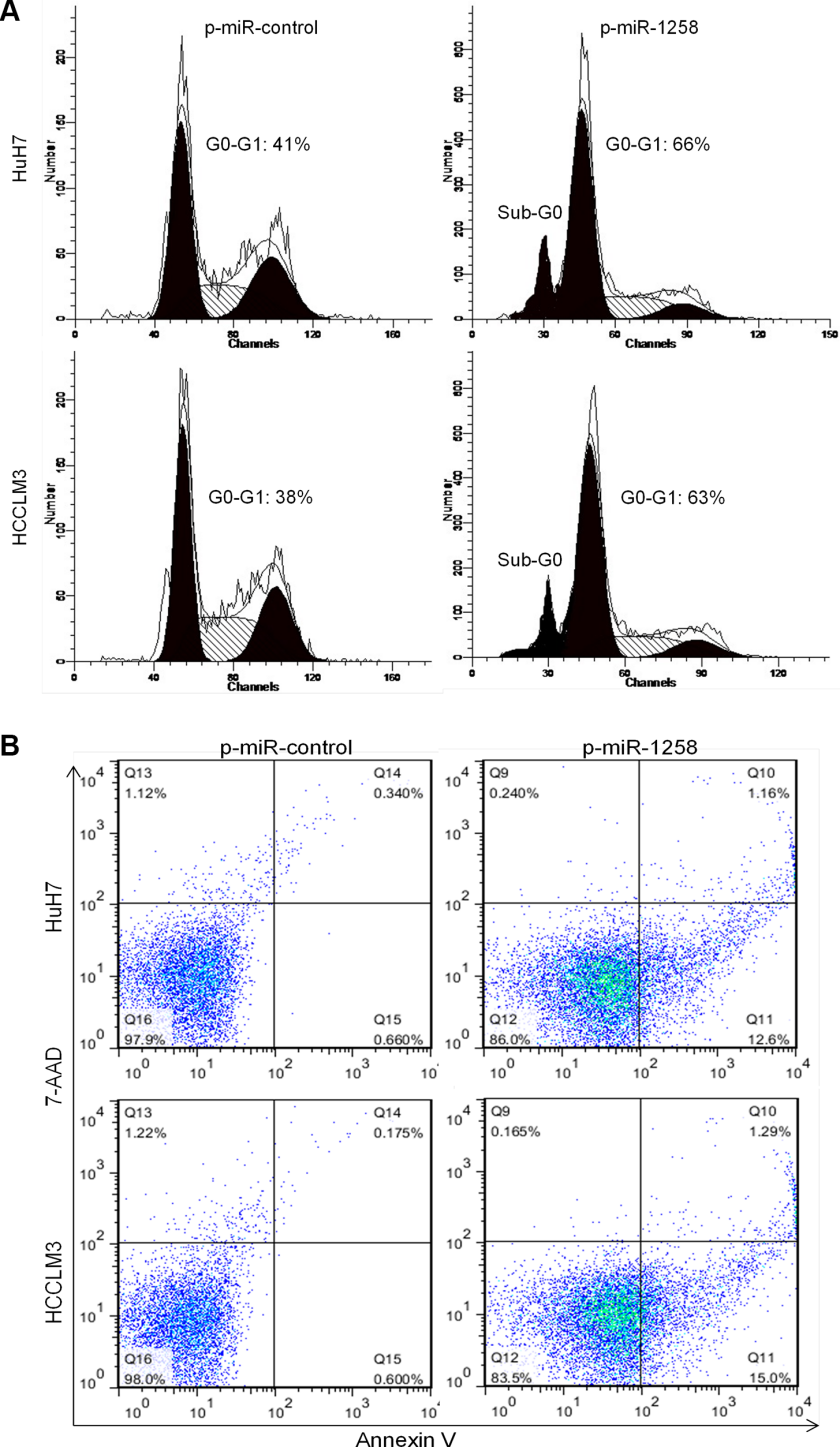
miR-1258 induces cell cycle arrest in G0/G1 phase and promotes cell apoptosis (**A**) Cell cycle distribution and apoptosis were evaluated by flow cytometry analysis performed 2 days after HuH7 and HCCLM3 cell transfected with p-miR-1258 or p-miR-control. Note that overexpression of miR-1258 induced cell cycle arrest in G0-G1 phase and dramatic increase in the percentage of cells with sub-G0 DNA content (indicative of apoptosis). (**B**) Representative images of Annexin V/7-AAD apoptotic assay showing that the percentage of Annexin V+ apoptotic cells was significantly increased in HuH7 and HCCLM3 cell transfected with p-miR-1258 in comparison to the p-miR-control transfected cells.

Cell cycle progression and apoptosis are two crucial causes of sustaining cell growth and proliferation. To further understanding the potential mechanisms by which overexpression of miR-1258 inhibits HCC cell growth and proliferation, we assessed cell cycle and apoptosis in HCC cell lines after miR-1258 overexpression. Flow cytometric analysis of cell cycle with propidium iodide (PI) in HuH7 and HCCL3 cells demonstrated that overexpression of miR-1258 led to a significant accumulation of cells at G0/G1-phase and a significant increase in cells in sub-G0, representing apoptotic cells (Figure 3A). Consistently, overexpression of miR-1258 also significantly increased the Annexin V+ cell population, which indicates the increase of cell apoptosis (Figure 3B). This data suggested that miR-1258 induced suppression of HCC cells growth and proliferation appeared to be correlated with increasing of cell cycle arrest in G0/G1 phase and cell apoptosis in HCC cells.

### miR-1258 suppresses cell migration and stemness to increase drug sensitivity

**Figure 4:**
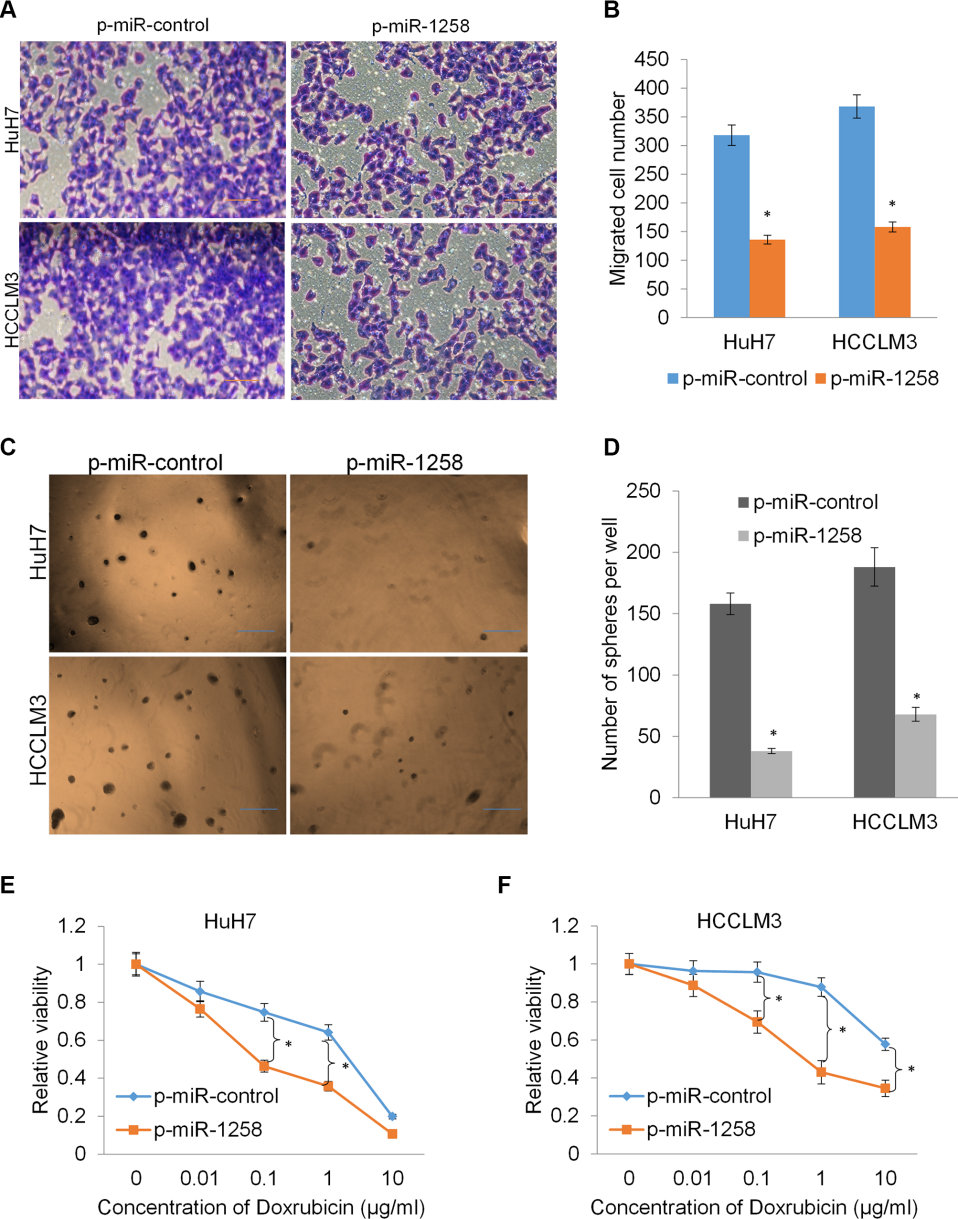
miR-1258 suppresses cell migration and stemness to increase drug sensitivity (**A** and **B**) The representative images (A) and number of migrated cells (B) in HuH7 and HCCLM3 cell transfected with p-miR-1258 or p-miR-control. (**C** and **D**) The representative images (C) and number of tumor spheres (D) in HuH7 and HCCLM3 cell transfected with p-miR-1258 or p-miR-control. (**E** and **F**) overexpression of miR-1258 sensitizes the HuH7 and HCCLM3 cell to doxorubicin by MTS analysis. All data are given as mean ± SD of three independent experiments. Significant differences are indicated, *P < 0.05.

Since our clinicopathological analysis also revealed that low expression of miR-1258 was associated with higher recurrence and metastasis potential (*P* < 0.05, Figure 1D and 1E), and the role of miR-1258 in the recurrence and metastasis of HCC has not been well characterized. We examined whether miR-1258 was a critical molecular on HCC cell migration by the transwell migration assay. As shown, overexpression of miR-1258 significantly suppressed the migration rates of HuH7 and HCCLM3 cells (Figure 4A and 4B). Increasing evidence suggested that the stemness of cancer cells is thought to be responsible for cancer initiation, recurrence and drug resistance. We next investigated the effects of miR-1258 on stemness and drug sensitivity of HCC cells. The tumor sphere formation results showed that overexpression of miR-1258 significantly inhibits sphere formation ability of HCC cells (Figure 4C and 4D). Since single-agent doxorubicin has been widely used to treat unresectable HCC, we then assayed the drug sensitivity to doxorubicin by comparing miR-1258 overexpressing cells and the according control cells. The results showed that overexpression of miR-1258 significantly contributed to increasing the sensitivity of HCC cells to doxorubicin (Figure 4E and 4F). These data collectively indicated that miR-1258 suppresses cell migration and stemness to increase drug sensitivity.

### CKS1B was identified as the critical downstream target of miR-1258

**Figure 5:**
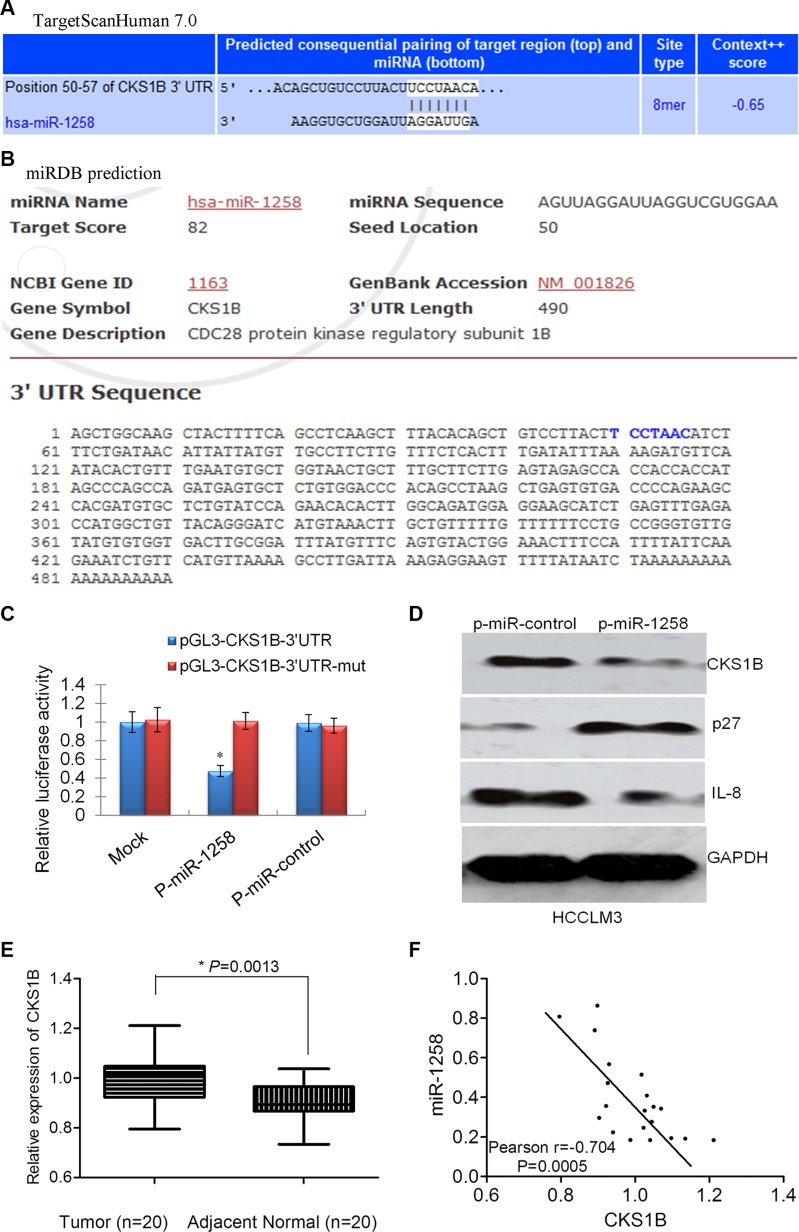
CKS1B was identified as the critical downstream target of miR-1258 (**A**) The binding site of CKS1B with miR- 1258 was predicted by TargetScanHuman 7.0 (A) or miRDB (**B**) (http://mirdb.org/miRDB/). (**C**) The normalized relative luciferase activity of HCCLM3 cells contransfected with p-miR-1258 or p-miR-control and pGL3- CKS1B-3′UTR wide type or pGL3- CKS1B-3′UTR-mutatant. (**D**) The representative images of western blot analysis showing that overexpression of miR-1258 inhibits the expression of CKS1B. The downstream p27 was increased while IL8 was decreased. (**E**) The normalized relative expression of CKS1B in 20 pairs of HCC and adjacent normal samples by RT-qPCR analysis. (**F**) The significant correlation between the expression of mR-1258 and CKS1B was observed in our tissue samples.

To understand the molecular mechanisms by which miR-1258 suppresses oncogenic properties of HCC cells, we used two different bioinformatics tools (TargetScan and miRDB) to predict putative target genes of miR- 1258. Among the 36 potential candidates predicted by TargetScan and miRDB (Table S1), CKS1B, CDC28 protein kinase regulatory subunit 1B, was further validated as the target of miR-1258. A predicted binding site for miR-1258 was identified in the 3′-UTR of CKS1B mRNA by TargetScan (Figure 5A) and miRDB (Figure 5B). To determine whether CKS1B is a direct target of miR- 1258, we constructed luciferase reporter plasmids using pGL3 plasmid encoding the wild-type 3′-UTR region of CKS1B (pGL3-CKS1B-3′UTR) or a mutated binding site of CKS1B 3′-UTR region (pGL3-CKS1B-3′UTR-mut). As shown in Figure 5C, overexpression of miR-1258 significantly decreased the relative luciferase activity of the CKS1B-WT-3′UTR reporter in HCC cells (*P* < 0.05), whereas the activity of the CKS1B-3′UTR-mut reporter was not affected by miR-1258. In addition, miR-1258 overexpression also significantly decreased the expression level of CKS1B protein in HCC cells (Figure 5D). Inhibition of CKS1B also contributes to increased expression of p27 and decreased expression of IL8, which are two important downstream targets of CKS1B [5]. qRT- PCR analysis showed that the expression of CKS1B was significantly overexpressed in HCC compared to adjacent normal samples (Figure 5E). The expression of CKS1B was negatively correlated with the expression of miR-1258 in HCC tissue samples (Figure 5F). The overexpression of CKS1B in HCC was also shown in TCGA dataset (Figure S1A). The expression correlation between CKS1B and p27 (CDKN1B), or CKS1B and IL8 in TCGA dataset was marginal or no correlation, but a significant negative correlation was also observed between miR-1258 and CKS1B in TCGA (Figure S1B–S1D). These data support that CKS1B was the critical downstream target of miR-1258.

### Re-expression of CKS1B overcomes miR-1258 induced apoptosis and increases stemness

**Figure 6:**
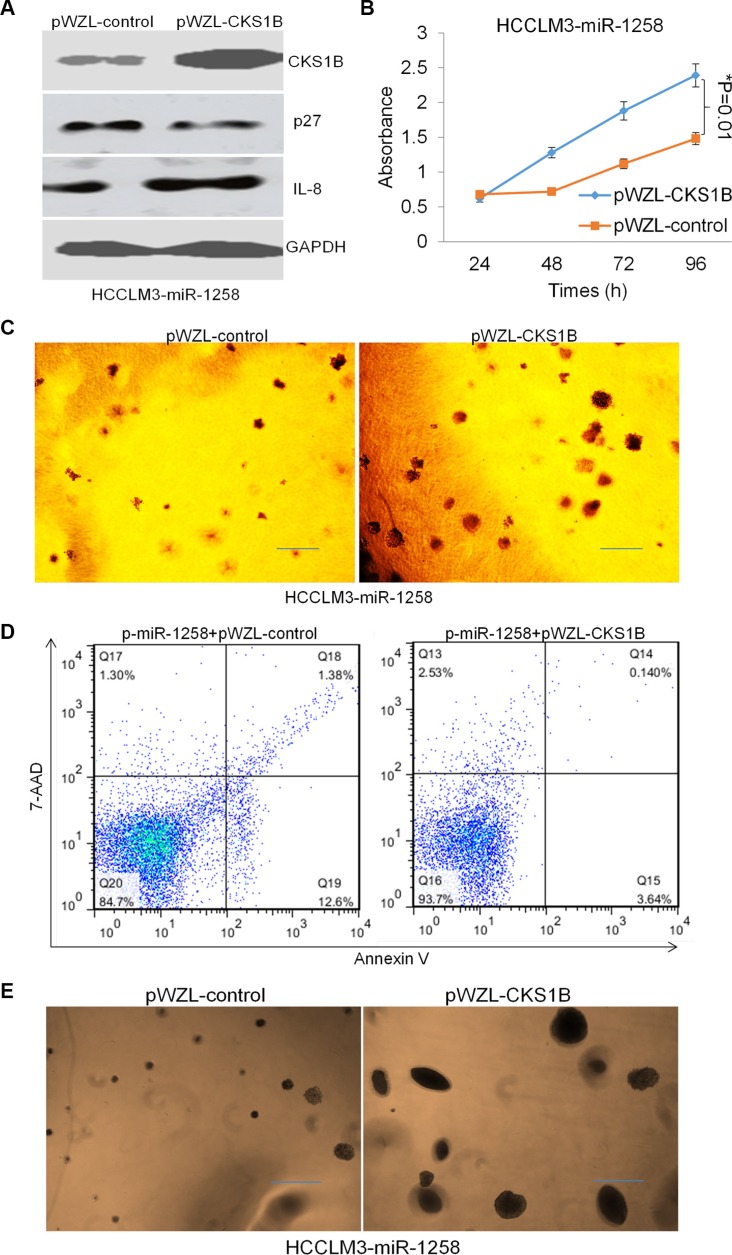
Re-expression of CKS1B overcomes miR-1258 induced apoptosis and increases stemness (**A**) The representative images of western blot analysis showing that re-expression of CKS1B inhibits p27 expression and promotes IL8 expression. (**B**) Re-expression of CKS1B significantly promotes liver cancer cells growth by MTS analysis. (**C**) The represent images of soft agar colony formation showing that re-expression of CKS1B promotes liver cancer cell growth. (**D**) Representative images of Annexin V/7-AAD apoptotic assay showing that the percentage of Annexin V+ apoptotic cells was significantly decreased by CKS1B overexpression. (**E**) The represent images of sphere formation showing that re-expression of CKS1B promotes liver cancer cell sphere formation ability.

To investigate whether miR-1258 exerted its effects by regulating CKS1B in HCC cells, we re- expressed the coding region sequences (CDS) of CKS1B in miR- 1258 transfected HCCLM3 cells using pWZL- CKS1B plasmid transfection. Western blot analysis was performed to confirm the overexpression effect of CKS1B levels (Figure 6A). Overexpression of CKS1B was also shown to decrease the expression of p27 and increase the expression of IL8. The MTS assay and soft agar colony formation assay results showed that overexpression of CKS1B promotes HCCLM3-miR-1258 stable cell growth and proliferation (Figure 6B and 6C). Meanwhile, Annexin V/7-AAD staining and flow cytometry analysis also showed that re-expression of CKS1B overcomes miR-1258 induced the increasing of Annexin V+ cell population, suggesting re-expression of CKS1B overcomes miR-1258 induced apoptosis (Figure 6D). The tumor sphere formation results also showed that re-expression of CKS1B significantly promotes sphere formation ability of miR-1258 transfected HCC cells. Re-expression of CKS1B induces much more and large tumor spheres (Figure 6E), suggesting re-expression of CKS1B increases stemness ability of miR-1258 transfected HCC cells. These data suggested that re-expression of CKS1B overcomes miR-1258 induced apoptosis and increases stemness of HCC cells.

### Stable overexpression of miR-1258 decreases tumorigenicity of HCC cells

**Figure 7:**
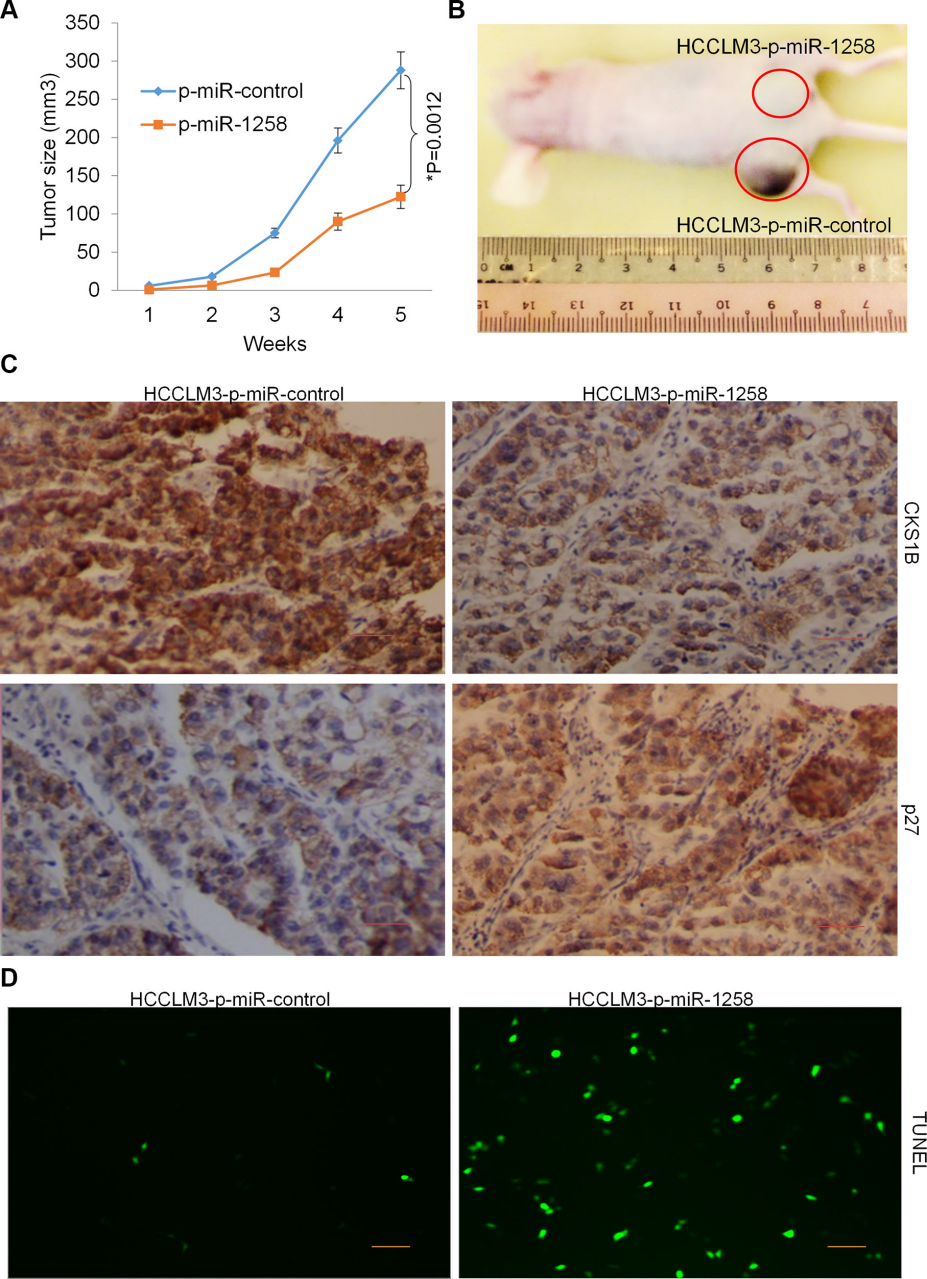
Stable overexpression of miR-1258 decreases tumorigenicity of HCC cells (**A**) Determination of the tumor growth. Tumor volume was calculated every week after injection. Data were mean ± SD of three independent experiments. *P = 0.0012. (**B**) Representative image of tumor formation. Nude mice were subcutaneously injected with 5 × 10^6^ P-miR-1258 or P-miR-control stable transfected HCCLM3 cells. (**C**) The Representative images of immunohistochemistry (IHC) analysis showing that the expression of CKS1B was significant decreased while p27 was significantly increased by overexpression of miR-1258. (**D**) Representative images of TUNEL staining of tumor tissues showing that overexpression of miR-1258 significantly promotes tumor cell apoptosis.

The effects of miR-1258 overexpression on the growth and proliferation of HCC cells were further confirmed by examining tumorigenicity *in vivo*. Immunodeficient BALB/C mice were subcutaneously injected with HCCLM3 cells that had been previously stable transfected with p-miR-1258 or p-miR-control. Throughout the tumorigenic period, the tumors formed from p-miR-1258-expressing HCCLM3 cells grew significantly slower than those formed from p-miR- control expressing cells (Figure 7A). After 35 days, the volume of the tumors generated from p-miR- 1258-expressing HCCLM3 cells were significantly smaller than those generated by p-miR-control-expressing cells (Figure 7B). The immunohistochemistry (IHC) staining of tumor tissues showed that the expression of CKS1B was significantly decreased, while the expression of p27 was significantly decreased in the tumor tissues formed from p-miR-1258-expressing HCCLM3 cells compared to those generated by p-miR-control-expressing cells (Figure 7C). The terminal deoxynucleotidyl transferase dUTP nick end labeling (TUNEL) assay also detect more TUNEL+ apoptosis cells in the tumor tissues formed from p-miR-1258-expressing HCCLM3 cells compared to those generated by p-miR-control-expressing cells (Figure 7D). These data suggested that stable overexpression of miR-1258 decreases tumorigenicity of HCC cells by inducing apoptosis.

## DISCUSSION

Increasing evidence has uncovered the contribution of miRNAs to pathogenesis of HCC because they can function as oncogenes or tumor suppressor genes. Certain miRNAs, called oncomiRs, play a causal role in the onset and maintenance of cancer when overexpressed. Tumors that depend on these microRNAs are regarded to display oncomiR addiction. Inhibition of oncomiRs using antisense oligomers (antimiRs) has been shown as an evolving therapeutic strategy [6]. Previous studies have demonstrated their potential value in the clinical management of patients with HCC as some miRNAs may be used as prognostic or diagnostic markers as well as potential therapeutic targets [7, 8]. For instance, systemic administration of miR-26a, a miRNA that is normally expressed at high levels in normal liver but decreased in HCC cells, in a mouse model of HCC using adeno-associated virus (AAV) results in inhibition of cancer cell proliferation, induction of tumor-specific apoptosis, and dramatic protection from disease progression without toxicity [9]. Moreover, the miR-26 expression status of HCC patients is associated with survival and response to adjuvant therapy with interferon alfa [10]. The administration of the liposomal formulated miR-34a mimic caused significant tumor growth inhibition in two different orthotopic liver cancer models and tumor regression was observed in more than one-third of the animals [11]. Therefore, understanding the critical role of miRNAs in HCC will provide a new tool for diagnostic, prognostic, and therapeutic potential.

In this study, we combined the miRNA expression profile of LIHC from TCGA and GSE36915. The downregulation of miR-1258 was consistently observed in both TCGA and GSE36915. However, the expression and critical role of miR-1258 in HCC have never been reported. Previous studies have showed that miR-1258 expression is attenuated in human breast cancer cells and patient tissues, and miR-1258 suppresses breast cancer brain metastasis by targeting heparanase (HSPE) [12, 13]. The miR-1258 has also been shown to inhibit the expression level of HPSE to influence the morbidity and metastasis of NSCLC. The miR-1258 is likely to become the key to the treatment of lung cancer metastasis [14]. Here, we demonstrated that miR-1258 was significantly downregulated in HCC tissues and that decreased miR-1258 expression was associated with worse clinic-pathological characteristics such as tumor recurrence and metastasis and a poor prognosis. miR-1258 overexpression inhibited cell growth, proliferation, cell cycle arrest, apoptosis, migration and stemness of HCC cells *in vitro* and tumorigenicity *in vivo*. These data suggested that miR-1258 may be a potential biomarker and therapeutic candidate of HCC.

Previous few studies identified that HPSE is a target of miR-1258 in breast [12, 13] and lung cancer [14]. However, there is no expression correlation between miR-1258 and HSPE in TCGA LIHC dataset, suggesting HSPE may not a critical target of miR-1258 in HCC (Figure S2). Further experiments indicated that miR-1258 directly targeted the CKS1B to suppress oncogenic properties of HCC cells. An inverse correlation between miR-1258 and CKS1B levels was found in HCC tissues. These results suggest that loss of miR-1258 may play an important role in the carcinogenesis of HCC with increasing of CKS1B. CKS1B has been shown overexpression in aggressive disease and regulated multiple myeloma growth and survival through SKP2- and p27Kip1-dependent and -independent mechanisms [15]. SKP2 and CKS1 have also been shown to promote degradation of cell cycle regulators and are associated with HCC prognosis [16]. The following study showed that CKS1B protein overexpression in HCCs is implicated in clinical aggressiveness but not in p27(Kip1) turnover, implying presence of p27(Kip1)-independent oncogenic attributes [17]. CKS1 supports hepatocarcinogenesis by NF-κB-mediated regulation of IL-8 expression, broadening the function of Cks1 in cancer beyond its role as a Skp2 cofactor in p27 ubiquitination [5]. Here, our study also showed that suppression of CKS1B by miR-1258 contributes to increased expression of p27 and decreased expression of IL8, suggesting both p27 and IL8 may play important role on mediating the critical role of miR-1258-CKS1B network in HCC. Taken together, these results demonstrate that loss of miR-1258 may be a diagnosis and prognostic biomarker and that the miR-1258-CKS1B axis is a potential therapeutic target in HCC.

## MATERIALS AND METHODS

### RNA isolation and RT-qPCR analysis

Total RNA from the tissue samples or cell lines was extracted using TRIzol reagent (Thermo Fisher Scientific, USA). The quality and quantity of isolated total RNA were assessed using the Agilent 2100 Bioanalyzer and NanoDrop ND-1000 Spectrophotometer (Agilent, Santa Clara, CA, USA). The qRT-PCR was performed as described [18] and the detail was descripted in the supporting information.

### Establish of stable cells and cell viability analysis

The HuH7 and HCCLM3 cell were seeded in six well plate and transfected with p-miR-1258 and p-miR- control plasmids (System Biosciences, USA). 48 hour after transfection, cells were trypsinized and seeded at 1: 10 dilution in 100 cm^2^ dishes. The puromycin (1 ug/ml for HuH7 and 3 ug/ml for HCCLM3) was added at 72 hours after transfection for selection. The stable cells clone were selected and examined by qRT-PCR. The cell viability was assessed by MTS [3-(4,5-dimethylthiazol-2-yl)-5-(3-carboxymethoxyphenyl)-2-(4-sulphophenyl)-2H- tetra zolium] assays using the CellTiter 96 AQueous One Solution Cell Proliferation Assay kit from Promega following the manufacturer's instructions. Each experiment was repeated three times.

### Flow-cytometry

The HuH7 and HCCLM3 cell were seeded in six well plates and transfected with p-miR-1258 and p-miR-control plasmids as mentioned above. 48 hour after transfection, the cells were collected for cell cycle and apoptosis analysis. The cell cycle and apoptosis was analysed by flow cytometry (FACSCanto II, BD Biosciences) using PI staining (for cell cycle) or Annexin V/7-AAD kits (for apoptosis) (BD Biosciences) according to the standard protocol.

### Luciferase reporter assay

The 3′-UTR sequence of CKS1B predicted to bind with miR-1258 or a mutated sequence within the predicted target sites was synthesized and inserted into the XbaI and FseI sites of the pGL3 control vector (Promega, Madison, WI). These constructs were named as pGL3-CKS1B-3′UTR or pGL3-CKS1B-3′UTR-mut, respectively. The detail of luciferase reporter assay was performed as descripted in the supporting information

### Animal studies, immunohistochemistry (IHC) and TUNEL assay

The established stable HCCLM3 cells were resuspended in PBS and implanted into the right and left flanks (5 × 10^6^ cells per flank) of male BALB/c nude mice via subcutaneous injections. Tumor volumes were determined each week by measuring their length (a) and width (b) using a vernier caliper. The tumor volume (V) was calculated according to the formula V = ab^2^/2. The statistical significance between tumor sizes in the P-miR-1258 and P-miR-control transfected groups was evaluated using the Student's *t* test. The tumors were fixed in 10% neutral buffered formalin before being processed into paraffin blocks. The tissue sections were stained with CKS1B and p27 (Abcam) using standard IHC techniques. TUNEL staining was performed using the DeadEnd fluorometric TUNEL system (Promega) according to the manufacturer's protocol. The number of TUNEL-positive cells was calculated under microscope.

## SUPPLEMENTARY MATERIALS FIGURES AND TABLE


