# Supplementary material for: Loss of miR-1258 contributes to carcinogenesis and progression of liver cancer through targeting CDC28 protein kinase regulatory subunit 1B
Source: Oncotarget. 2016 May 30;7(28):43419–31. doi: 10.18632/oncotarget.9728 (PMC5190034; doi:10.18632/oncotarget.9728)
Supplement: Supplementary file 1 [file oncotarget-07-43419-s001.pdf]

Loss of miR-1258 contributes to carcinogenesis and progression of liver cancer through targeting CDC28 protein kinase regulatory subunit 1B

Supplementary Materials

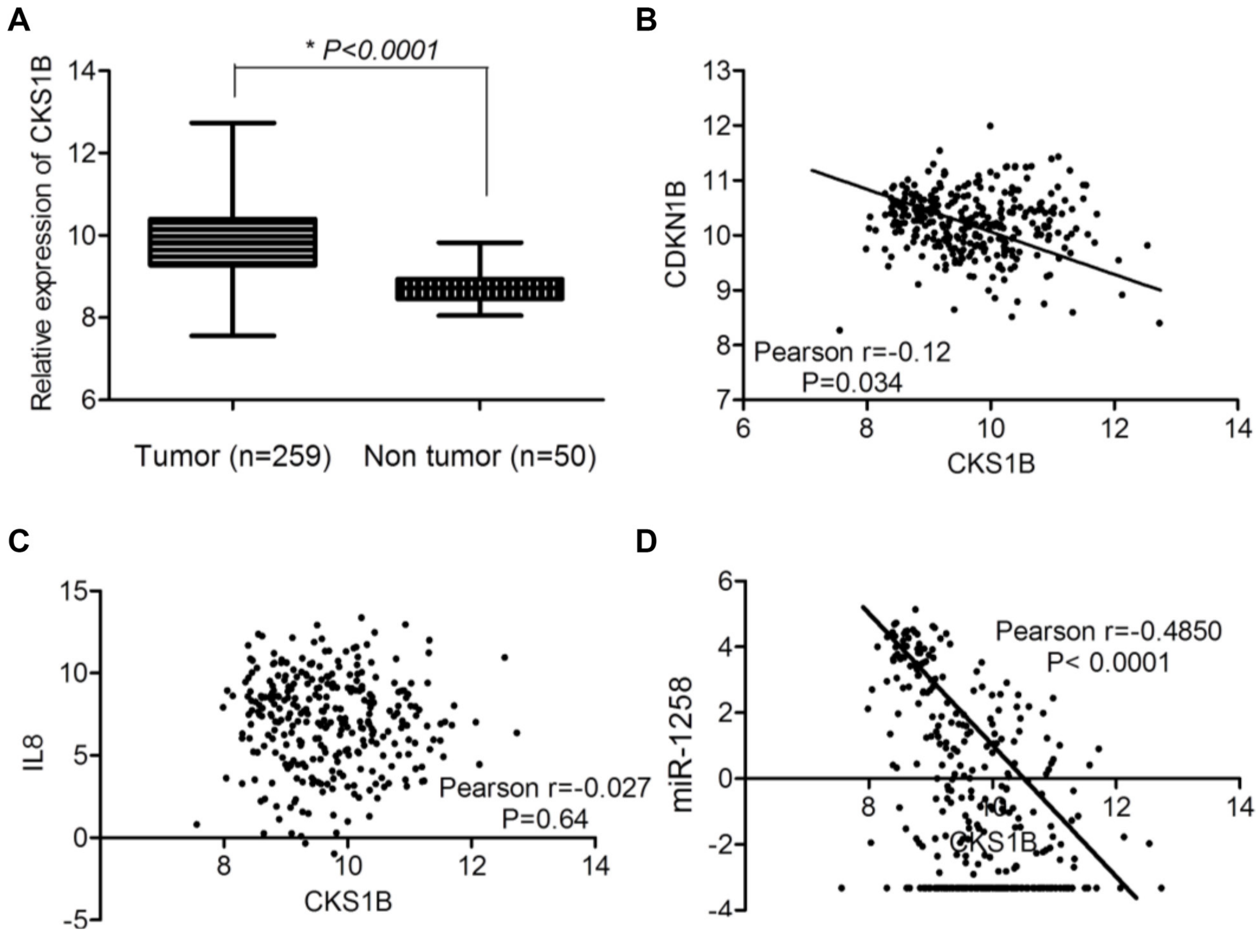

Supplementary Figure S1: The expression of CKS1B in TCGA dataset (A) and the expression correlation between CKS1B and p27 (CDKN1B) (B), or and IL8 (C) and miR-1258 in TCGA dataset (D).

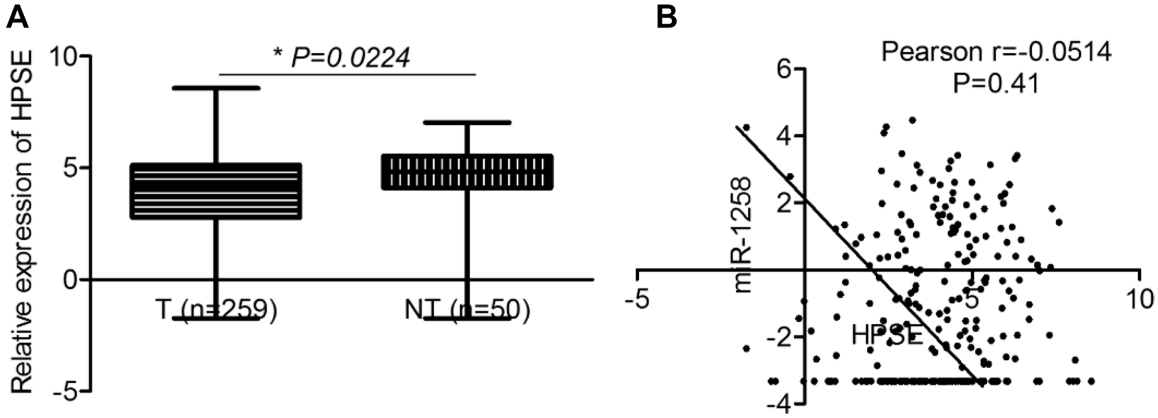

Supplementary Figure S2: The expression of HPSE in TCGA dataset (A) and the expression correlation between miR-1258 and HPSE in TCGA dataset (B).

Supplementary Table S1: The predict putative target genes of miR- 1258 by two different bioinformatics tools (target scan and miRDB)

Target Rank	Target Score	miRNA Name	Gene Symbol	Gene Description	Representative transcript	Gene name	Number of 3P-seq tags supporting UTR + 5	Link to sites in UTRs	Site counts	Cumulative weighted context++ score	Total context++ score
125	60	hsa-miR-1258	ANXA3	annexin A3	ENST00000264908.6	annexin A3	3305	Sites in UTR	1	–0.55	–0.55
89	65	hsa-miR-1258	ATG12	autophagy related 12	ENST00000500945.2	autophagy related 12	1822	Sites in UTR	3	–0.52	–1.25
26	84	hsa-miR-1258	BNIP3L	BCL2/adenovirus E1B 19kDa interacting protein 3-like	ENST00000380629.2	BCL2/adenovirus E1B 19kDa interacting protein 3-like	191	Sites in UTR	2	–0.56	–0.67
140	58	hsa-miR-1258	C2orf66	chromosome 2 open reading frame 66	ENST00000342506.2	chromosome 2 open reading frame 66	22	Sites in UTR	1	–0.41	–0.56
118	62	hsa-miR-1258	C9orf47	chromosome 9 open reading frame 47	ENST00000375851.2	chromosome 9 open reading frame 47	5	Sites in UTR	2	–0.56	–0.56
32	82	hsa-miR-1258	CKS1B	CDC28 protein kinase regulatory subunit 1B	ENST00000308987.5	CDC28 protein kinase regulatory subunit 1B	186	Sites in UTR	1	–0.65	–0.65
29	82	hsa-miR-1258	CLIC4	chloride intracellular channel 4	ENST00000374379.4	chloride intracellular channel 4	226	Sites in UTR	1	-0.48	-0.48
13	90	hsa-miR-1258	CSDC2	cold shock domain containing C2, RNA binding	ENST00000306149.7	cold shock domain containing C2, RNA binding	31	Sites in UTR	2	–0.61	–0.61
9	93	hsa-miR-1258	EMX2	empty spiracles homeobox 2	ENST00000442245.4	empty spiracles homeobox 2	54	Sites in UTR	1	–0.52	–0.57
130	59	hsa-miR-1258	FAM206A	family with sequence similarity 206, member A	ENST00000322940.6	family with sequence similarity 206, member A	266	Sites in UTR	2	–0.74	–0.74
44	77	hsa-miR-1258	FAM84A	family with sequence similarity 84, member A	ENST00000295092.2	family with sequence similarity 84, member A	69	Sites in UTR	2	–0.47	–0.74
50	75	hsa-miR-1258	FAR2	fatty acyl CoA reductase 2	ENST00000536681.3	fatty acyl CoA reductase 2	193	Sites in UTR	1	–0.55	–0.61
105	63	hsa-miR-1258	FASLG	Fas ligand (TNF superfamily, member 6)	ENST00000340030.3	Fas ligand (TNF superfamily, member 6)	5	Sites in UTR	1	–0.43	–0.43
153	55	hsa-miR-1258	GAPT	GRB2-binding adaptor protein, transmembrane	ENST00000396776.2	GRB2-binding adaptor protein, transmembrane	5	Sites in UTR	2	–0.58	–0.58
73	69	hsa-miR-1258	GDPD1	glycerophosphodiester phosphodiesterase domain containing 1	ENST00000284116.4	glycerophosphodiester phosphodiesterase domain containing 1	138	Sites in UTR	1	–0.46	–0.5
28	83	hsa-miR-1258	GNG2	guanine nucleotide binding protein (G protein), gamma 2	ENST00000556752.1	guanine nucleotide binding protein (G protein), gamma 2	199	Sites in UTR	1	–0.49	–0.55

57	73	hsa-miR-1258	HDDC3	HD domain containing 3	ENST00000559898.1	HD domain containing 3	1127	Sites in UTR	1	–0.61	–0.62
79	68	hsa-miR-1258	HERPUD2	HERPUD family member 2	ENST00000396081.1	HERPUD family member 2	30	Sites in UTR	1	–0.46	–0.46
98	64	hsa-miR-1258	HOXD10	homeobox D10	ENST00000249501.4	homeobox D10	182	Sites in UTR	1	–0.44	–0.44
41	79	hsa-miR-1258	LDHAL6A	lactate dehydrogenase A-like 6A	ENST00000280706.2	lactate dehydrogenase A-like 6A	5	Sites in UTR	1	–0.56	–0.56
139	58	hsa-miR-1258	MAPRE3	microtubule-associated protein, RP/EB family, member 3	ENST00000233121.2	microtubule-associated protein, RP/EB family, member 3	303	Sites in UTR	1	–0.43	–0.43
8	93	hsa-miR-1258	PCNP	PEST proteolytic signal containing nuclear protein	ENST00000296024.5	PEST proteolytic signal containing nuclear protein	944	Sites in UTR	1	–0.45	–0.46
135	59	hsa-miR-1258	PDF	peptide deformylase (mitochondrial)	ENST00000288022.1	peptide deformylase (mitochondrial)	190	Sites in UTR	1	-0.5	-0.5
68	70	hsa-miR-1258	PDYN	prodynorphin	ENST00000539905.1	prodynorphin	5	Sites in UTR	1	-0.56	-0.56
42	78	hsa-miR-1258	PLCD4	phospholipase C, delta 4	ENST00000450993.2	phospholipase C, delta 4	11	Sites in UTR	1	–0.45	–0.45
24	85	hsa-miR-1258	PMP22	peripheral myelin protein 22	ENST00000395938.2	peripheral myelin protein 22	1152	Sites in UTR	1	–0.5	-0.5
12	91	hsa-miR-1258	PRLR	prolactin receptor	ENST00000342362.5	prolactin receptor	95	Sites in UTR	4	–0.58	–0.75
10	92	hsa-miR-1258	RYBP	RING1 and YY1 binding protein	ENST00000477973.2	RING1 and YY1 binding protein	342	Sites in UTR	2	–0.45	–0.47
56	73	hsa-miR-1258	SERPINB7	serpin peptidase inhibitor, clade B (ovalbumin), member 7	ENST00000398019.2	serpin peptidase inhibitor, clade B (ovalbumin), member 7	37	Sites in UTR	1	–0.51	–0.51
3	97	hsa-miR-1258	SGMS1	sphingomyelin synthase 1	ENST00000361781.2	sphingomyelin synthase 1	100	Sites in UTR	2	–0.64	–0.65
109	63	hsa-miR-1258	SMIM17	small integral membrane protein 17	ENST00000598409.1	small integral membrane protein 17	5	Sites in UTR	1	–0.63	–0.63
103	63	hsa-miR-1258	THEMIS	thymocyte selection associated	ENST00000368250.1	thymocyte selection associated	5	Sites in UTR	2	–0.65	–0.65
74	69	hsa-miR-1258	TMBIM1	transmembrane BAX inhibitor motif containing 1	ENST00000258412.3	transmembrane BAX inhibitor motif containing 1	2201	Sites in UTR	2	–0.71	–0.71
76	69	hsa-miR-1258	TMEM68	transmembrane protein 68	ENST00000523073.1	transmembrane protein 68	77	Sites in UTR	1	–0.61	–0.66
19	86	hsa-miR-1258	UBE2H	ubiquitin-conjugating enzyme E2H	ENST00000355621.3	ubiquitin-conjugating enzyme E2H	855	Sites in UTR	3	–0.68	–0.79
35	81	hsa-miR-1258	ZNF100	zinc finger protein 100	ENST00000358296.6	zinc finger protein 100	142	Sites in UTR	1	–0.43	–0.43

MATERIALS AND METHODS

Cell culture and tissue samples

Liver cancer cells (Hep3B, HuH7, HepG2, HCCLM3, Mahlavu, SNU475) were cultured in Dulbecco's modified Eagle's medium with 10% FBS and 100 units/mL of penicillin and 100 μg/mL of streptomycin (Invitrogen, Carlsbad, CA). All cell lines were maintained at 37°C in the presence of 5% CO_2. The collection of tumor and adjacent normal liver tissues from HCC patients were approved by the Ethics Committee of the Yijishan Hospital of Wannan Medical College and the Affiliated Cancer Hospital of Zhengzhou University. None of the patients had received chemotherapy or radiotherapy before surgical resection. All specimens were immediately frozen in liquid nitrogen and stored at –80°C until analysis.

qRT-PCR analysis

For mRNA detection, the total RNA was reverse transcribed using the SuperScript® VILO™ cDNA Synthesis Kit (Invitrogen, CA). The qPCR was performed using SsoFast™ EvaGreen® Supermix (Bio-Rad). For miRNA detection, the total RNA samples were polyadenylated and reverse transcribed for a two-step quantitative RT-PCR reaction using the NCode™ VILO™ miRNA cDNA Synthesis Kit and EXPRESS SYBR® GreenER™ miRNA qRT-PCR Kits (Invitrogen, CA) according to the manufacturer's instructions. The *HPRT1* or *U6* gene was used as an endogenous control, and fold changes were calculated via relative quantification ($2^{-\Delta Ct}$).

Soft agar colony assay

The established p-miR-1258 and p-miR-control stable transfected cells or pWZL-CKS1B and pWZL-control transfected cells was mixed with tissue culture medium containing 0.6% low-melting-point agarose (Sigma Sant Louise, MO), resulting in a final agar concentration of 0.3%. Then, 500 µL of the cell suspension (800 cells) was immediately plated in 24-well plates coated with 500 µL 0.6% agar in tissue culture medium and cultured at 37°C with 5% CO2. The plates were kept in the incubator and the number of colonies formed was counted under an inverted light microscope (×40 objective) after 2–3 weeks. The assay was analyzed in duplicate in three independent experiments.

In vitro transwell cell migration assay

Cell migration was detected using 24 well Transwell chambers without Matrigel matrix (8 µm pore size, BD Biosciences, CA, USA). In brief, 600 µl complete medium was added to the bottom chamber, stable transfected cells were suspended in serum-free medium, and 500 µl of the cell suspension (containing 5×10^4 cells) was placed in the upper chamber. After 24 hours, the cells on the top surface of the membrane were mechanically removed using a cotton swab, and the cells on the bottom surface of the membrane were fixed in 95% ethanol and stained with a 4 g/L crystal violet solution. Cells adhering to the bottom surface of the membrane were counted in five randomly selected areas under a 40× microscope field. Each experiment was repeated three times.

Sphere formation assay

Single stably p-miR-1258 and p-miR-control stable transfected cells or pWZL-CKS1B and pWZL-control transfected cells (1×10^4) were plated onto a 6-well ultra-low attachment plate (Corning, Corning, NY) in serum-free DMEM-F12, supplemented with 2 mM L-glutamine, 1% sodium pyruvate (Invitrogen), 1% MEM nonessential amino acids (Invitrogen), 1% insulin-transferrin-selenium-X supplement (Invitrogen), 1 µM dexamethasone (Wako), 200 µM L-ascorbic acid 2-phosphate (Sigma-Aldrich), 10 mM nicotinamide (Wako), 100 µg/ml penicillin, and 100 U/ml streptomycin supplemented with 20 ng/ml epithelial growth factor and 10 ng/ml fibroblast growth factor-2 (Invitrogen). After 2–3 weeks of culture, the number of spheres (diameter > 40 µm) was manually counted in three randomly selected fields at the magnification of 40× using an inverted fluorescence microscope. Results were obtained from three independent experiments performed in triplicate.

Luciferase reporter assay

For the reporter assay, HCCLM3 cells were plated onto 24-well plates and transfected with the above constructs and P-miR-1258 or P-miR-control vectors using Lipofectamine 3000 (Thermo Fisher Scientific, USA). A Renilla luciferase vector pRL-SV50 (Promega, Madison, WI) was co-transfected to normalize the difference in the transfection efficiency. After 48 h, the cells were harvested and assayed using the dual-luciferase reporter assay system (Promega, Madison, WI) according to the manufacturer's instructions. Results were obtained from three independent experiments performed in duplicate.

Survival and statistical analysis

The experimental data are presented as the mean ± standard deviation (SD). All statistical analyses were performed using ANOVA or a two-tailed Student's *t* test by GraphPad Prism 5.0 (GraphPad Software, La Jolla, CA). The survival curves were created using the Kaplan-Meier method and statistically compared using a log-rank test. Differences were considered statistically significant when the *P*-values were less than 0.05.
